# The nervous system of the adult ascidian *Ciona intestinalis* Type A (*Ciona robusta*): Insights from transgenic animal models

**DOI:** 10.1371/journal.pone.0180227

**Published:** 2017-06-26

**Authors:** Tomohiro Osugi, Yasunori Sasakura, Honoo Satake

**Affiliations:** 1 Bioorganic Research Institute, Suntory Foundation for Life Sciences, Kyoto, Japan; 2 Shimoda Marine Research Center, University of Tsukuba, Shizuoka, Japan; NORWAY

## Abstract

The nervous system of ascidians is an excellent model system to provide insights into the evolutionary process of the chordate nervous system due to their phylogenetic positions as the sister group of vertebrates. However, the entire nervous system of adult ascidians has yet to be functionally and anatomically investigated. In this study, we have revealed the whole dorsal and siphon nervous system of the transgenic adult ascidian of *Ciona intestinalis* Type A (*Ciona robusta*) in which a *Kaede* reporter gene is expressed in a pan-neuronal fashion. The fluorescent signal of Kaede revealed the innervation patterns and distribution of neurons in the nervous system of *Ciona*. Precise microscopic observation demonstrated the clear innervation of the anterior and posterior main nerves to eight and six lobes of the oral and atrial siphons, respectively. Moreover, visceral nerves, previously identified as unpaired nerves, were found to be paired; one nerve was derived from the posterior end of the cerebral ganglion and the other from the right posterior nerve. This study further revealed the full trajectory of the dorsal strand plexus and paired visceral nerves on either side from the cerebral ganglion to the ovary, and precise innervation between the cerebral ganglion and the peripheral organs including the gonoduct, cupular organ, rectum and ovary. The differential innervation patterns of visceral nerves and the dorsal strand plexus indicate that the peripheral organs including the ovary undergo various neural regulations. Collectively, the present anatomical analysis revealed the major innervation of the dorsal and siphon nervous systems of adult *Ciona*.

## Introduction

The biological functions and organization of the central nervous system are correlated with the evolutionary lineages of animals. Recent molecular phylogenetic analyses indicated that tunicates (ascidians, thaliaceans, and appendicuralians) constitute the sister group of vertebrates [1–4]. The structure and functions of the central and peripheral nervous system of the larva of a cosmopolitan ascidian, *Ciona intestinalis*, have been investigated including the number and types of neurons, axon projections, neuronal circuit, and development of the nervous system [5–11], revealing the morphological and developmental similarity of the central nervous system between *Ciona* larva and other chordates [6,9–13]. While considerably less attention has been paid to the central nervous system of adult ascidians [10,14], approximately forty neuropeptides and peptide hormones have been identified in *Ciona* adults over the past fifteen years [15–23]. These studies provided evidences that the central nervous system of adult *Ciona* possesses much more homologs or prototypes of vertebrate neuropeptides and hormones than protostome model organisms such as *Caenorhabditis elegans* and *Drosophila melanogaster* [15–23]. During metamorphosis from the larva to the infant, *Ciona* reconstructs the central nervous systems that regulate basal biological functions including food intake, digestion, absorption, and reproduction [24,25]. Indeed, Ci-TK, a *Ciona* homolog of a vertebrate neuropeptide, tachykinin, was shown to be secreted to the ovary from the central nervous system to specifically enhance the growth of vitellogenic follicles [23,26,27]. Altogether, these findings shed light on the biological significance of the nervous system of adult ascidians as a basal model for the essential functions and evolutionary process of the chordate nervous system that controls fundamental biological behaviors.

The nervous system of adult *Ciona* is composed of the cerebral ganglion, neural gland, anterior and posterior nerves, visceral nerves, dorsal strand, and dorsal strand plexus [24]. In these components, the cerebral ganglion, dorsal strand plexus and visceral nerves form the dorsal nervous system that is thought to regulate a wide variety of biological events including food intake, excretion, and reproduction [24]. In juvenile *Ciona*, a recent study revealed the number and localization of cholinergic, glutamatergic, and GABAergic/glycinergic neurons in the cerebral ganglion, and axonal trajectories of cholinergic neurons [28]. In other classes of tunicates, the nervous system was investigated based on the serotonin-like immunoreactivity in the thaliaceans and appendicuralians in a whole-mount preparation [29,30]. The detailed morphology of the secondary sensory cells was also investigated, and their structures were compared among tunicate species [31,32]. Moreover, the development of the central nervous system of thaliaceans was analyzed, and the central nervous systems in embryo and larvae were compared among tunicate species and other chordates [33]. However, the nervous system of adult ascidians has been neither structurally nor functionally analyzed due to the difficulties in investigating the innervation and trajectory by conventional histological techniques [24,34].

Prohormone convertase 2 (PC2) is a major protease responsible for the endoproteolytic maturation of peptide hormones or neuropeptides, and the *PC2* gene is evolutionarily conserved and expressed in most neurons including those of *Ciona* [35,36]. Hence, the transgenic *Ciona* harboring a reporter gene construct driven by the *PC2* promoter exhibits reporter gene expression in a pan-neuronal fashion. The visualization of neurons in the transgenic line can lead to the precise elucidation of the entire neural network.

In this study, we show major innervation of the dorsal and siphon nervous system of transgenic adult *C*. *intestinalis* Type A (recently recognized as *Ciona robusta* [37]) that expresses the *Kaede* reporter gene driven by a *PC2* gene promoter. The whole structure of the dorsal and siphon nervous system of adult ascidians will pave the way to understanding of the entire neural network of *Ciona* and the evolutionary processes of the nervous system in chordates.

## Materials and methods

### Transgenic lines

*Ciona intestinalis* Type A (*Ciona robusta* [37]) was used to create transgenic lines. We called *Ciona intestinalis* Type A as *Ciona* in this manuscript in accordance with the description on the database of transgenic lines of the National BioResource Project of Japan (http://marinebio.nbrp.jp/). The *PC2>Kaede* lines of *Ciona*, Tg[MiCiPC2K]2 and Tg[MiCiPC2K]3, were created by *Minos*-mediated transgenesis [38,39] and provided from the National BioResource Project. These lines were cultured using the inland culture system as described previously [40]. Adult transgenic animals were used in this study. The two transgenic lines, Tg[MiCiPC2K]2 and Tg[MiCiPC2K]3, showed similar expression patterns of Kaede in the nervous system. We observed the general expression pattern of Kaede of three individuals of each transgenic line, and confirmed that all transgenic *Ciona* showed essentially identical Kaede expression. The transgenic lines are indicated at the upper right of each figure.

### Tissue preparation

Animals were anesthetized using L-menthol based on the previous method [41]. In brief, 0.56% (weight per volume) L-menthol (Nacalai Tesque, Kyoto, Japan) in ethanol was prepared and used as a stock solution. The stock solution was further diluted by 1% (volume per volume) in artificial sea water before use. Animals were soaked into the diluted L-menthol solution for 10 min. Tissues were dissected under the fluorescence stereo microscope (Leica M205 FA; Leica Microsystems, Wetzlar, Germany) and fixed with 4% paraformaldehyde in PBS at 4 C° overnight. The fixed tissues were soaked in 2 mg/ml glycine in PBS for quenching paraformaldehyde. The tissues were washed three times with PBS and used for morphological analyses.

### Whole-mount observation of tissues

After removal of the tunic, fixed tissues were placed in a dish with a rubber sheet or a slide glass chamber filled with PBS (AGC TECHNO GLASS, Tokyo, Japan) and fixed with needles as appropriate. A fluorescence stereo microscope (Leica M205 FA; Leica Microsystems) and image acquisition software (Leica AF6000E; Leica Microsystems) were used for low magnification observation. A confocal microscope with a 10× or 20× objective (FLUOVIEW FV1000; Olympus, Tokyo, Japan) was used for high magnification observation and three-dimensional images acquisition. To construct three-dimensional images, a z-stack function was used and 200–300 cross section images of fixed tissues from top to bottom per sample were collected. The focus interval was 1μm for each section image. Names of the nerves were based on the previous study with partial modification [42]. The general morphological descriptions were based on the previous study [43]. The term "innervation" is used to explain the distribution of nerves but does not include their functions.

### Freeze sectioning

Fixed tissues were embedded in Super Cryoembedding Medium-L1 (Leica Microsystems Japan, Tokyo, Japan) and serially sectioned at a 10 μm thickness with a CryoStar NX70 cryostat (Thermo Fisher Scientific Inc., Waltham, MA, USA) at ‒18°C. The sections were placed onto FRONTIER-coated slides (FRC-04; Matsunami Glass Ind., Ltd., Osaka, Japan), and then mounted with Fluoromount (Diagnostic BioSystems, Pleasanton, CA, USA). Confocal microscope with 10×, 20×, or 40× objective (FLUOVIEW FV1000; Olympus) was used for high magnification observation.

## Results and discussions

### Overview of the Kaede-positive innervation of the transgenic adult *Ciona*

**Fig 1:**
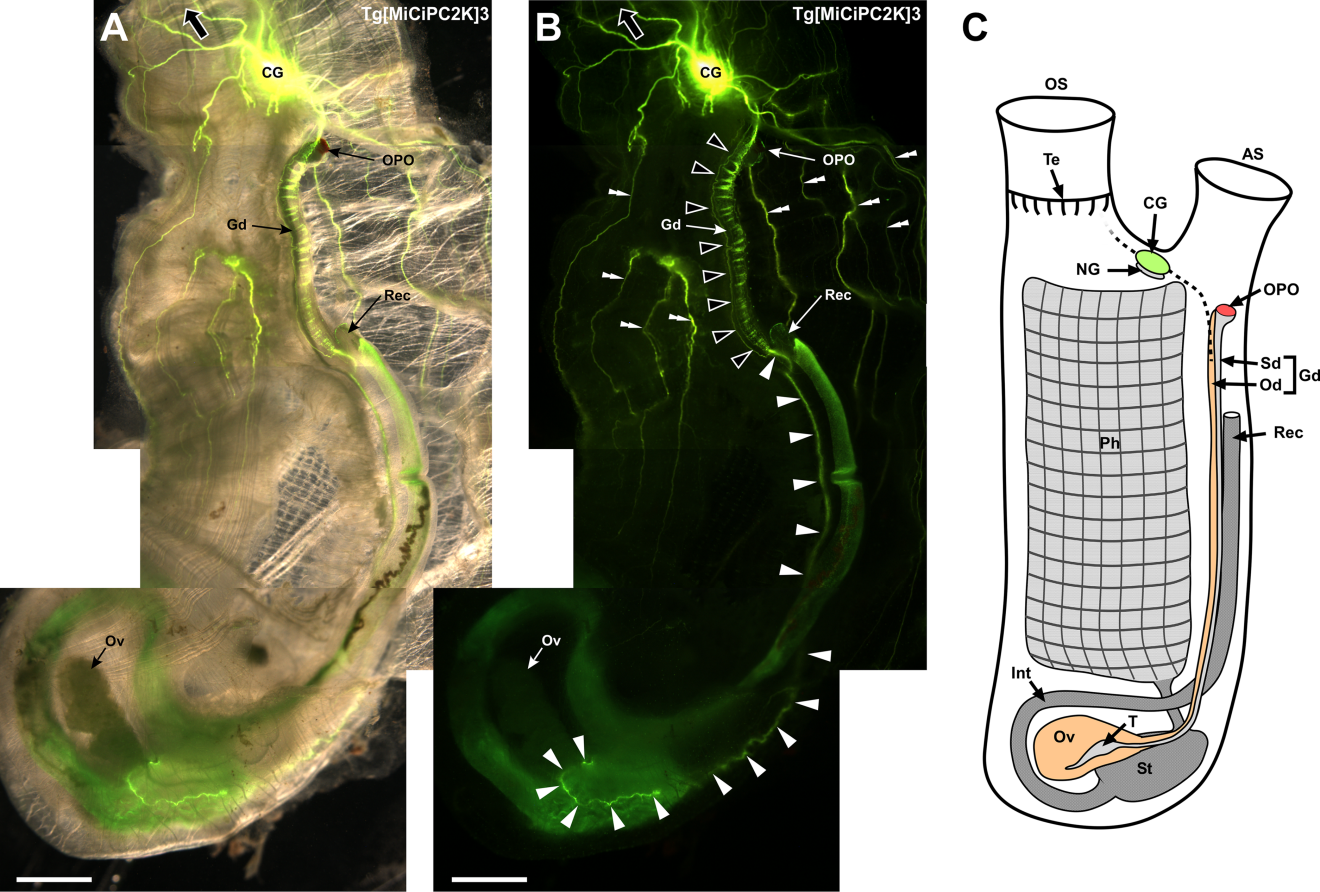
**(A) Superimposed image of the dorsal view of the transgenic *Ciona*.** Major organs locating at the dorsal region and Kaede-positive nerves are shown. Four images taken by the fluorescence stereo microscope were merged. A large arrow indicates the direction of the oral siphon. The atrial siphon was removed to better visualize the cerebral ganglion and the orange-pigmented organ (OPO). **(B) Dark field image of the dorsal view of the transgenic *Ciona*.** Four images taken by the fluorescence stereo microscope were merged. A large arrow indicates the direction of the oral siphon. The trajectory from the cerebral ganglion to the ovary is indicated by open and closed arrowheads. Note that the banded pattern in the trajectory (open arrowheads) was caused by the contraction of the body muscle. Nerves to the body muscles are indicated by double arrowheads. **(C) Schematic of an adult *Ciona*.** The key anatomical parts of an adult *Ciona* are indicated. AS, atrial siphon; CG, cerebral ganglion; Gd, gonoduct; Int, intestine; NG, neural gland; Od; oviduct, OPO, orange-pigmented organ; OS, oral siphon; Ov, ovary; Ph, pharynx; Rec, rectum; Sd, spermiduct; St, stomach; T, testis; Te, tentacle. Scale bars indicate 2.5 mm.

The transgenic *Ciona* strongly expressed Kaede fluorescent protein in the nervous system throughout the body. The Kaede-positive nerve connections were visualized among the cerebral ganglion, siphons and body muscles (Fig 1A, 1B and 1C). Furthermore, Kaede-positive neural structure lied between the cerebral ganglion and the ovary *via* the orange-pigmented organ (OPO), gonoduct and rectum (Fig 1A, 1B and 1C). These anatomical views prompted us to investigate the innervation in a whole-mount preparation of the transgenic adult *Ciona* in greater detail. We focused on the innervation of the dorsal and siphon nervous system, which had been poorly understood.

### Innervation of anterior and posterior nerves to the siphons

**Fig 2:**
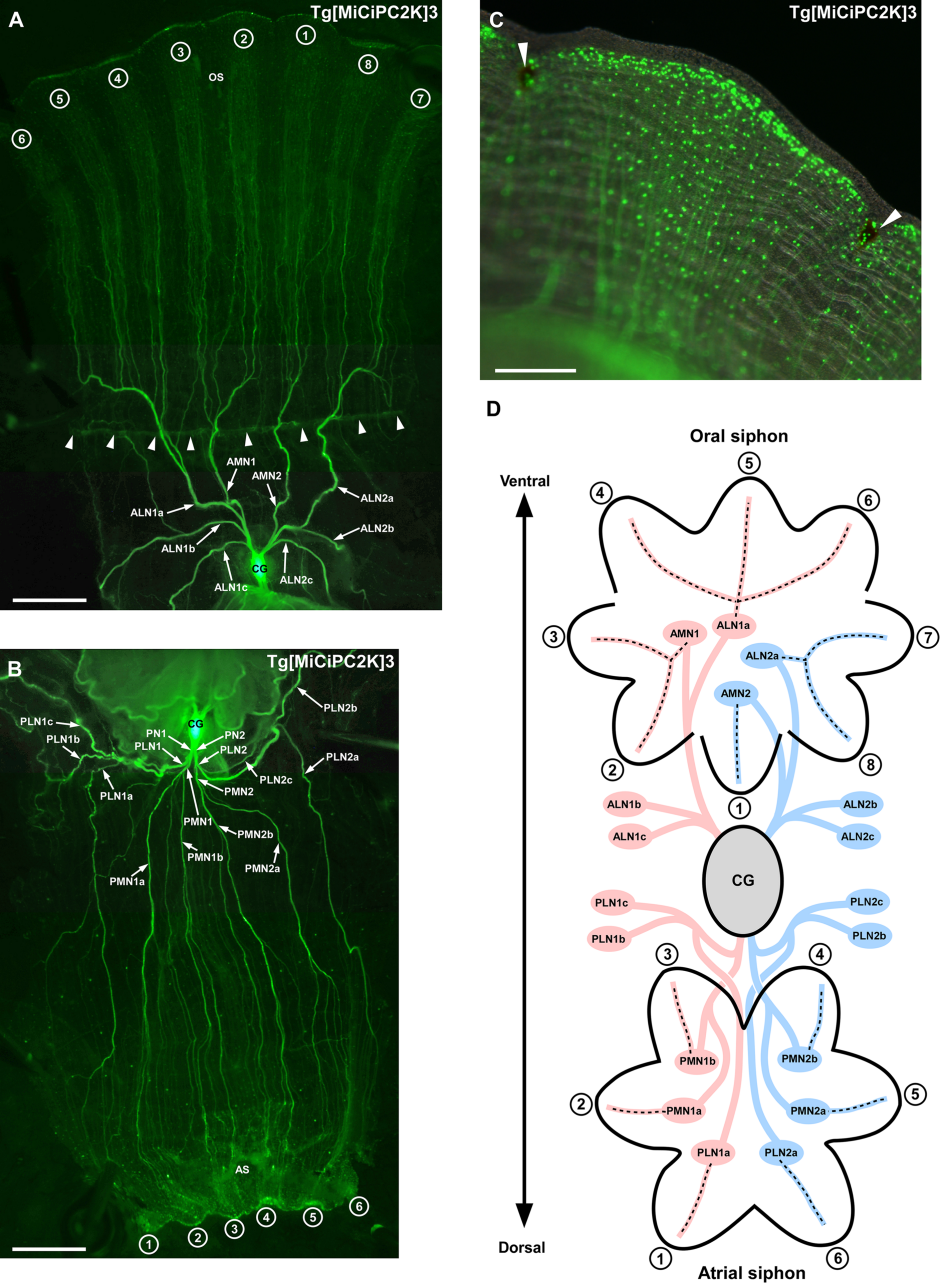
**(A) Innervation of the anterior nerves to the oral siphon and periphery.** Anterior nerves are indicated by arrows. Arrowheads indicate tentacle row. The lobes of the oral siphons are numbered from 1 to 8. **(B) Innervation of the posterior nerves to the atrial siphon and periphery.** Posterior nerves are indicated by arrows. The lobes of the atrial siphons are numbered from 1 to 6. **(C) Magnified image of the siphon lobe.** The innervation of nerves to the edge of the siphon lobe is shown. Pigment organs are indicated by arrowheads. The small dots are Kaede-positive cells. **(D) Schematic illustration of the anterior and posterior innervation to the siphons.** The illustration is the overhead view of the oral and atrial siphons. The numbers of the lobes correspond to those in (A) and (B). Nerves derived from the left part of the cerebral ganglion are indicated in pink. Nerves derived from the right part of the cerebral ganglion are indicated in blue. The innervations are indicated by dotted lines. All images were taken by the fluorescence stereo microscope. AMN, anterior medial nerve; ALN, anterior lateral nerve; PMN, posterior medial nerve; PLN, posterior lateral nerve; CG, cerebral ganglion; OS, oral siphon; AS, atrial siphon. Scale bars indicate 2.5 mm in A and B and 500μm in C.

Several early studies demonstrated the innervation of anterior nerves to the oral siphon and posterior nerves to the atrial siphon in ascidians including *Ciona* [24,44]. However, the precise number of nerves and their innervation patterns remained to be elucidated. The present study clearly showed that eight main anterior nerves and ten main posterior nerves are derived from the cerebral ganglion and innervate the oral siphon, atrial siphon, and periphery branching into thin nerves (Fig 2A and 2B). These nerves innervated the edge of the siphon lobes and pigment organs (Fig 2C), suggesting that they regulate the movement and/or sensory functions of siphons. Interestingly, the eight lobes of the oral siphon were found to be innervated by four different anterior nerves. Three ventral lobes (numbered by 4 to 6 in Fig 2D) and two left-lateral lobes (numbered by 2 and 3 in Fig 2D) were innervated by the anterior lateral nerve 1a (ALN1a) and anterior medial nerve 1 (AMN1), respectively, derived from the left region of the cerebral ganglion (Fig 2A and 2D). In contrast, one dorsal lobe (numbered by 1 in Fig 2D) and two right-lateral lobes (numbered by 7 and 8 in Fig 2D) were innervated by the anterior medial nerve 2 (AMN2) and anterior lateral nerve 2a (ALN2a), respectively, derived from the right region of the cerebral ganglion (Fig 2A and 2D). The body walls were innervated by the other four anterior main nerves, ALN 1b, 1c, 2b, and 2c (Fig 2A and 2D). Minor neural connections were seen between these nerves and the oral siphon and between these nerves and tentacles (Fig 2A). These innervation patterns to the oral siphon suggest that the lobes of the oral siphon are grouped into four histological units according to the projected nerves: three ventral lobes, left two and right two lateral lobes, and one dorsal lobe.

Each of six lobes of the atrial siphon was innervated by a single nerve of six posterior main nerves, posterior lateral nerve 1a (PLN1a), PLN2a, posterior medial nerve 1a, (PMN1a), PMN1b, PMN2a and PMN2b, and peripheral body walls were innervated by the other four posterior main nerves, PLN1b, 1c, 2b and 2c (Fig 2B and 2D). These results revealed an outstanding difference in the innervation patterns between the oral and atrial siphons. The aforementioned innervation of major nerves was observed in all transgenic lines, while the number and shape of fiber projection was found to vary among individuals (S1 Fig). Thus, the innervation of major nerves is conserved as a basal nervous system in *Ciona*.

We observed efferent peptidergic innervations from the cerebral ganglion to the siphons in the previous immunohistochemical study [45]. In addition, afferent synapses were dominantly found in the tentacles of siphons in *Ciona* [31]. Afferent innervations of the peptidergic neurons in the siphons were also reported in other ascidian species *Corella inflata* [46]. These findings will contribute to identification of major efferent and afferent nerves in the Kaede-positive nervous system.

*Ciona* individuals have eight siphon lobes and pigment organs located between lobes [47]. All of the oral siphon lobes and pigment organs were regenerated in their original forms when the distal tip or middle part of the oral siphon was removed [48]. On the other hand, the number of regenerated lobes and pigmented organ was disordered when the siphon was removed at the base part below the tentacle row, suggesting that the proximal-distal axis determines the regeneration pattern of the oral siphon [48]. Notably, this study showed that the corresponding base part is innervated by ALN1a, ALN2a, AMN1, and AMN2 that are divided into eight branches above the tentacle row and further projects numerous nerves to the oral lobes and pigment organs (Fig 2A, 2C and 2D). Our findings allow us to hypothesize that these nerves participate in the proximal-distal axis-directed normal regeneration of the oral siphon lobes and pigment organs.

### The visceral nerves and dorsal strand plexus around the cerebral ganglion

**Fig 3:**
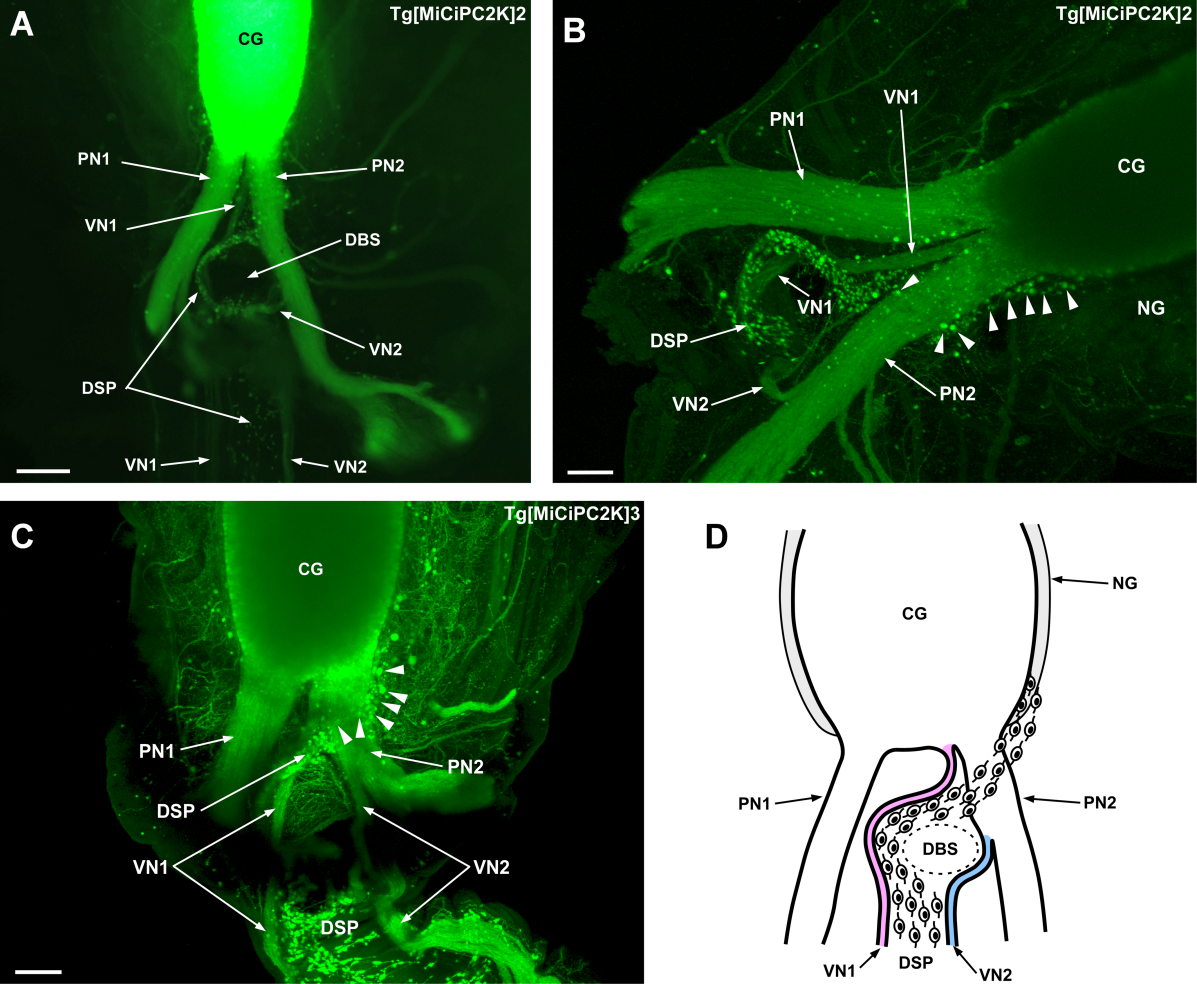
**(A) Front view of the posterior cerebral ganglion, nerves and dorsal strand plexus.** The overview of the posterior nerves (PN1 and PN2), two visceral nerves (VN1 and VN2), and dorsal strand plexus are shown. **(B) Side view of the posterior cerebral ganglion, nerves, and dorsal strand plexus.** The neurons of the dorsal strand plexus emerging between the cerebral ganglion and the neural gland passing above the PN2, are indicated by arrowheads. **(C) Front view of the posterior cerebral ganglion, nerves, and dorsal strand plexus.** The trajectory of VN1 and VN2 on both sides of the dorsal strand plexus are shown. Indicated by arrowheads are the neurons of the dorsal strand plexus emerging between the cerebral ganglion and the neural gland and passing above PN2. **(D) Schematic illustration of the front view of the cerebral ganglion, nerves, and dorsal strand plexus.** The left visceral nerve (VN1) is indicated in pink and the right visceral nerve (VN2) is indicated in blue. Low magnification image (A) was obtained by the fluorescence stereo microscope. High magnification and three-dimensional images (B and C) were obtained by the confocal laser scanning microscope. The fluorescent signal was not seen in the neural gland, suggesting that *PC2* promoter is inactive. CG, cerebral ganglion; PN, posterior nerve; VN, visceral nerve; DSP, dorsal strand plexus; NG, neural gland; DBS, dorsal blood sinus. Scale bars indicate 200μm in (A) and 100μm in (B) and (C).

Little has been known about innervation patterns between the cerebral ganglion and the ovary through the dorsal strand plexus and the visceral nerves [15,24,49–51]. In the previous studies, the visceral nerve was identified as an unpaired nerve in *Ciona* [13,52–54]. In contrast, we identified “paired” visceral nerves. The visceral nerve 1 (VN1) originated from the cerebral ganglion adjacent to the right posterior nerve 2 (PN2), and visceral nerve 2 (VN2) branched from PN2, both of which extended to the periphery along with either side of the dorsal strand plexus (Fig 3A–3D). The observation from the serial sections also confirmed the presence of the two visceral nerves arising from the posterior cerebral ganglion and PN2 (S2 Fig). VN1 and VN2 were derived from the right part of the posterior cerebral ganglion, suggesting that the right half of the cerebral ganglion regulates VN1 and VN2. VN1 that is derived from the posterior cerebral ganglion corresponds to the previously reported visceral nerve [13, 52–54], and VN2 that is derived from PN2 is newly reported in this study. In the immunohistochemical studies of gonadotropin-releasing hormone (GnRH), only a single visceral nerve was observed [52,53]. In other studies using transgenic lines *CiPhox2*::*YFP* [11] or E[MiTSAdTPOG]15 line [54] that expresses a fluorescent protein in nervous tissues also reported a single visceral nerve. It is highly likely that all nerves were not visualized and VN2 might have not been observed by immunohistochemistry or morphological analyses of transgenic ascidians in these studies, particularly, due to the difficulty in distinguishing VN2 from other nerves that also branch from PN2 as shown in Fig 3B. In addition, fibrous tissues lie above the branching point of VN2 and surgical removal of this tissues was quite difficult (S3 Fig). Altogether, the present study provides evidence that *Ciona* is endowed with paired visceral nerves.

This study also revealed entire trajectories of the visceral nerves and the dorsal strand plexus from the cerebral ganglion to the peripheral organs. Furthermore, we identified the origin of the dorsal strand plexus in detail with spatial information in a three-dimensional view. The neurons constituting the dorsal strand plexus emerged between the cerebral ganglion and neural gland, and passed above PN2 and curved around the dorsal blood sinus, and built the dorsal strand plexus (Fig 3B and 3C). The neural gland is not a nervous tissue [24, 34] and Kaede fluorescent signal was not seen in the neural gland (Fig 3B), suggesting that the neurons innervating to the dorsal strand plexus are derived from the ventral region of the cerebral ganglion.

### Innervation of the dorsal strand plexus and visceral nerves to the orange-pigmented organ

**Fig 4:**
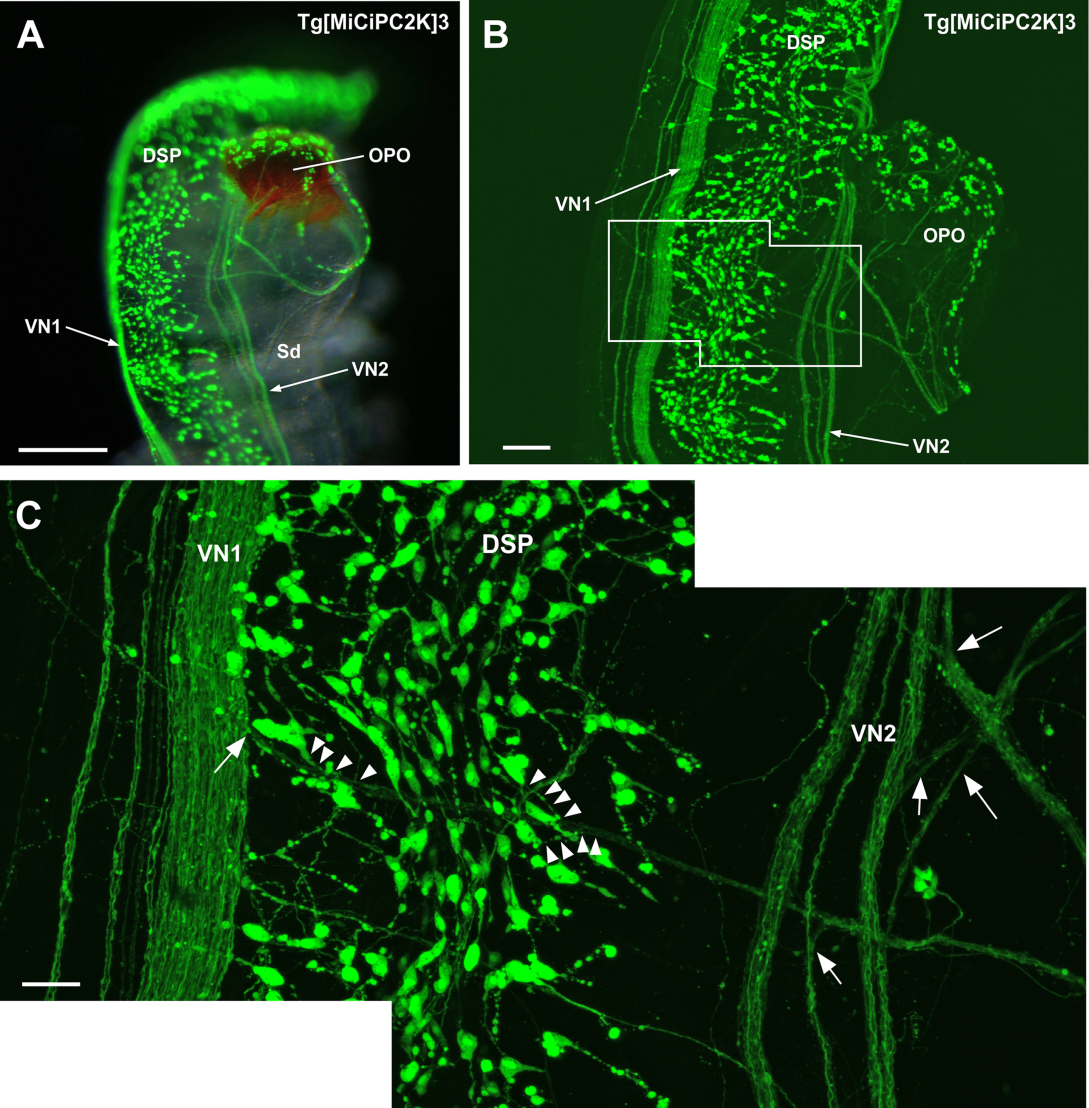
**(A) Image of the dorsal strand plexus, visceral nerve and orange-pigmented organ.** The image was obtained by the fluorescence stereo microscope. VN1, VN2, DSP and innervation to the orange-pigmented organ (OPO) are shown. **(B) Image of the dorsal strand plexus, visceral nerve and orange-pigmented organ.** The image was obtained by the confocal laser scanning microscope. Dominant innervation from VN2 and minor innervation from VN1 to OPO are shown. **(C) Magnified image of the framed region in (B).** The branching points of the visceral nerves to the OPO are indicated by arrows. The neurons and axons innervating to the OPO are indicated by arrowheads. DSP, dorsal strand plexus; OPO, orange-pigmented organ; Sd, spermiduct; VN, visceral nerve. Scale bars indicate 250μm in (A), 100μm in (B) and 30μm in (C).

Neurons of the dorsal strand plexus built a broad band-like structure and passed near the orange-pigmented organ (OPO) located at the end of spermiduct (Fig 4A). Two visceral nerves, VN1 and VN2, were observed on either side of the dorsal strand plexus (Fig 4A). Interestingly, numerous nerves from VN2 innervated OPO, compared with only a few VN1 nerves (Fig 4B and 4C). Kaede-positive neurons and their axons were also observed on top of OPO (Fig 4A and 4B). These results are largely consistent with the previous study using other transgenic lines [55]. Interestingly, the axons from OPO neurons mainly projected to VN2 (Fig 4B and 4C). Together, these innervation patterns suggest both afferent and efferent trajectories between OPO and VN2. In addition, OPO is known as a duct for sperm release [55], suggesting the regulation of the sperm release by VN2. Some neurons in the dorsal strand plexus also projected to OPO (Fig 4C), suggesting their neural communication. These observations are in part consistent with the previous studies on the distribution of GnRH immunoreactive neurons in the dorsal strand plexus [15,50,52,56]. In the present study, however, a greater number of neurons were found in the dorsal strand plexus than the GnRH-immunoreactive neurons, suggesting other neuropeptidergic neurons in the dorsal strand plexus.

**Fig 5:**
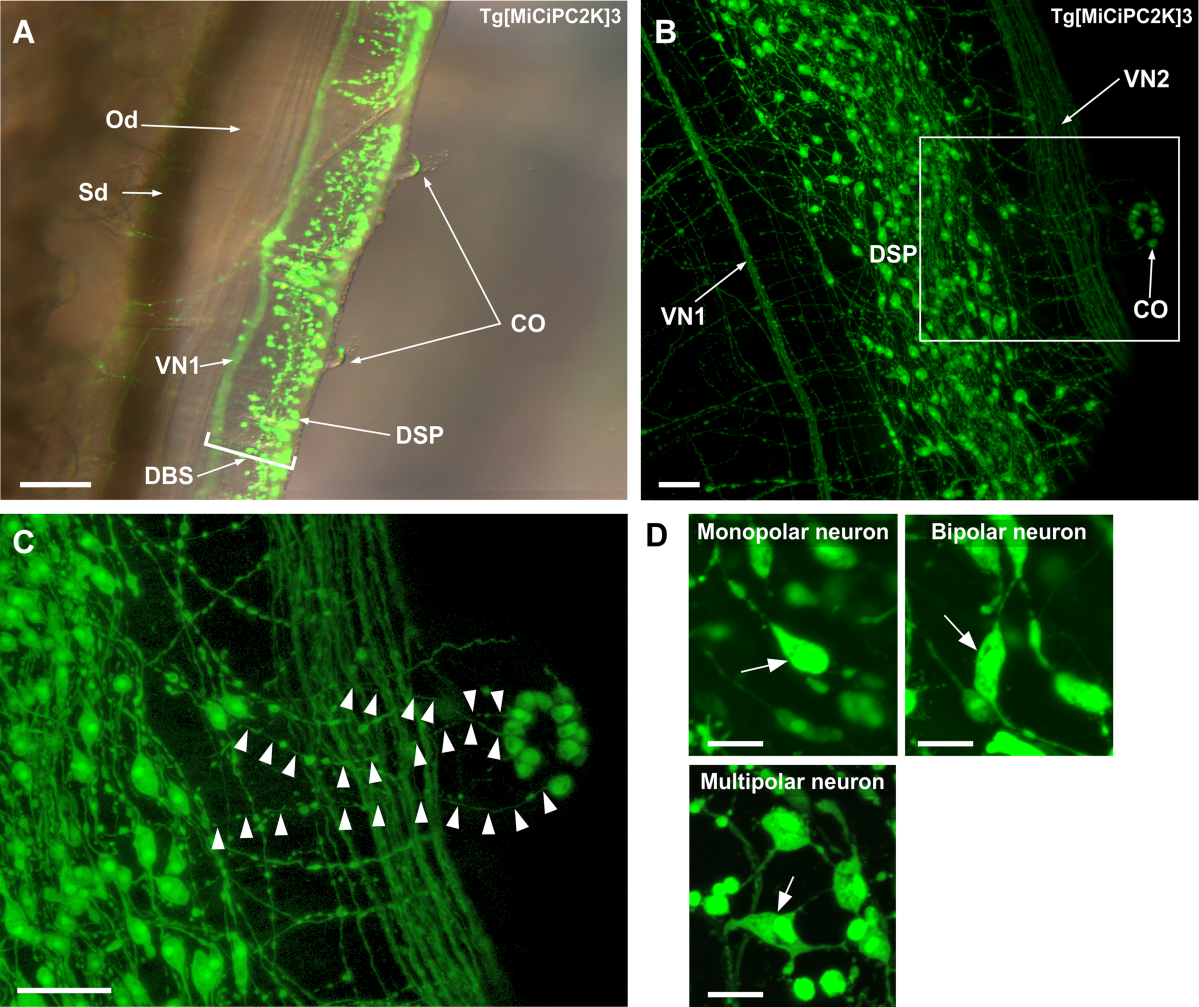
**(A) Side view of the dorsal strand plexus and visceral nerve.** The image was obtained by the fluorescence stereo microscope as a superimposed image. Spatial relationship of the dorsal strand plexus, visceral nerve, cupular organ, spermiduct and oviduct are shown. Hair sensory cells in the cupular organ are also visible. **(B) Image around the dorsal strand plexus.** The image was obtained by the confocal laser scanning microscope. Two visceral nerves (VN1 and VN2), neurons in the dorsal strand plexus, and neurons in the cupular organ are shown. **(C) Magnified image of the framed region in (B).** The axons of the cupular organ neurons are indicated by arrowheads. **(D) Magnified image of the neurons in the dorsal strand plexus in Tg[MiCiPC2K]3 line.** The image was obtained by the confocal laser scanning microscope. The monopolar, bipolar and multipolar neurons are indicated by arrows. VN, visceral nerve; DBS, dorsal blood sinus; DSP, dorsal strand plexus, Od, oviduct; Sd, spermiduct; CO, cupular organ. Scale bars indicate 200μm in (A), 40μm in (B) and (C), and 15μm in (D).

Subsequently, we observed innervation of neurons to the site located posterior to OPO. The dorsal strand plexus, VN1, and VN2 run along with the dorsal blood sinus located adjacent to the oviduct and spermiduct (Fig 5A). Moreover, Fig 5B–5D demonstrates the distribution of numerous monopolar, bipolar, and multipolar neurons in the dorsal strand plexus, neurons in the cupular organ, and nerves of VN1 and VN2. Furthermore, neurons in the cupular organ were found to project their axons to the neurons in the dorsal strand plexus, VN1, and VN2 (Fig 5C). The cupular organ is a putative hydrodynamic sensor consisting of neurons and sensory cells in *Ciona* [55,57]. Combined with these findings, the present observation suggests that the dorsal strand plexus and visceral nerves are involved in the transmission of sensory information from cupular organs to the cerebral ganglion.

### Innervation of the dorsal strand plexus and visceral nerves to the rectum

**Fig 6:**
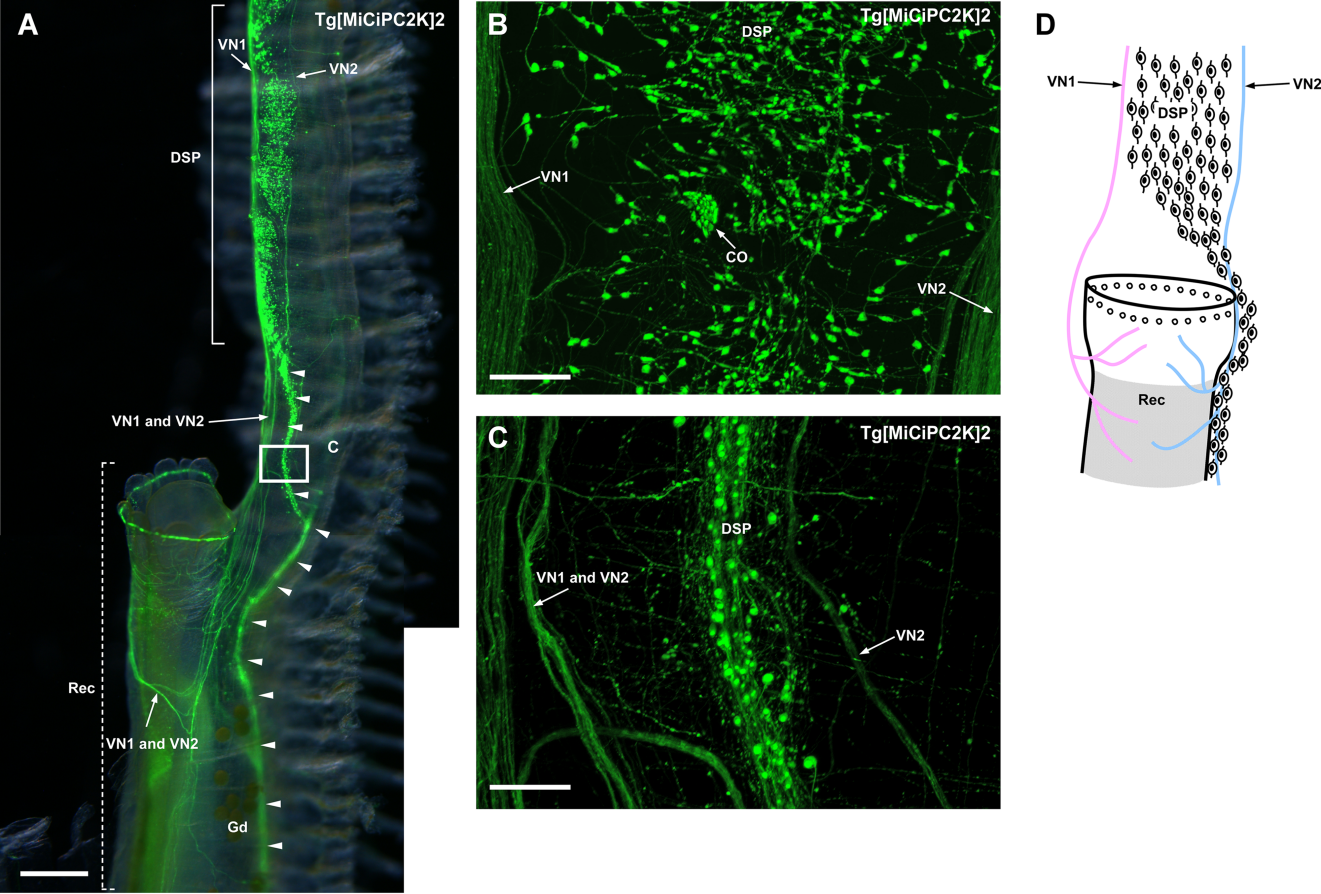
**(A) Image of the rectum, dorsal strand plexus and visceral nerves.** The image was obtained by the fluorescence stereo microscope. The anterior part of the rectum is shown and indicated by the dotted lines. Innervations of the VN1 and VN2 to the rectum are shown. The dorsal strand plexus becomes thinner around the rectum and continues to the periphery along with the rectum and gonoduct (arrowheads). **(B) Magnified image of the dorsal strand plexus above the rectum** The image was obtained by the confocal laser scanning microscope. The dorsal strand plexus is large in width above the rectum and 20–30 neurons were laterally distributed. **(C) Magnified image of the rectangle region in (A)** The image was obtained by the confocal laser scanning microscope. The dorsal strand plexus becomes thinner and less than ten neurons were laterally distributed. **(D) Schematic illustration of the innervation of the visceral nerves and dorsal strand plexus to the rectum.** Neurons in the dorsal strand plexus are shown as neuron-shaped illustrations. Kaede-positive cells at the edge of the rectum are shown as open circles. VN1 and VN2 are shown in pink and blue, respectively. The region where Kaede-positive small cells are distributed is shadowed. CO, cupular organ; DSP, dorsal strand plexus; Gd, gonoduct; Rec, rectum; VN, visceral nerve. Scale bars indicate 750μm in (A) and 100μm in (B) and (C).

Nerves from VN1 innervated the rectum and Kaede-positive cells at the edge of the rectum, suggesting VN1 regulate the rectum (Fig 6A).

Nerves from VN2 and/or the dorsal strand plexus also projected to the rectum (Fig 6A). Nerves from VN2 extended to the periphery along with the dorsal strand plexus (Fig 6A and 6D). The dorsal strand plexus became thinner near the rectum (Fig 6A, 6C and 6D). 20–30 neurons were laterally distributed in the dorsal strand plexus above the rectum (Fig 6B), while less than ten neurons were distributed in the thinner dorsal strand plexus (Fig 6C). Unlike the upper part of the dorsal strand plexus located above the rectum (Fig 6B), the cupular organs were not seen in the lower part of the dorsal strand plexus (Fig 6C). The absence of the cupular organs suggests that the lower part of the dorsal strand plexus is not involved in sensory transmission, although the possibility cannot be excluded that other sensory neurons, not contained in the cupular organs, exist in these regions (e.g. proprioceptive neurons [46]).

Numerous Kaede-positive small cells were also found in the rectum, and these cells were widely distributed to the dorsal region of the rectum (Fig 6A). Although innervation to other regions of the rectum than the posterior edge awaits further study, such localization of Kaede-positive small cells suggests some neuronal regulations of the rectum. Compared with the previous study [24], the present study revealed the structural organization of the nervous system around the rectum. GnRH-immunoreactive axons were found to poorly innervate the rectum [52]. Therefore, other neuropeptides and neurotransmitters than GnRH are likely to regulate the functions of the rectum via the dorsal strand plexus and visceral nerves.

### Innervation of the dorsal strand plexus to the ovary

**Fig 7:**
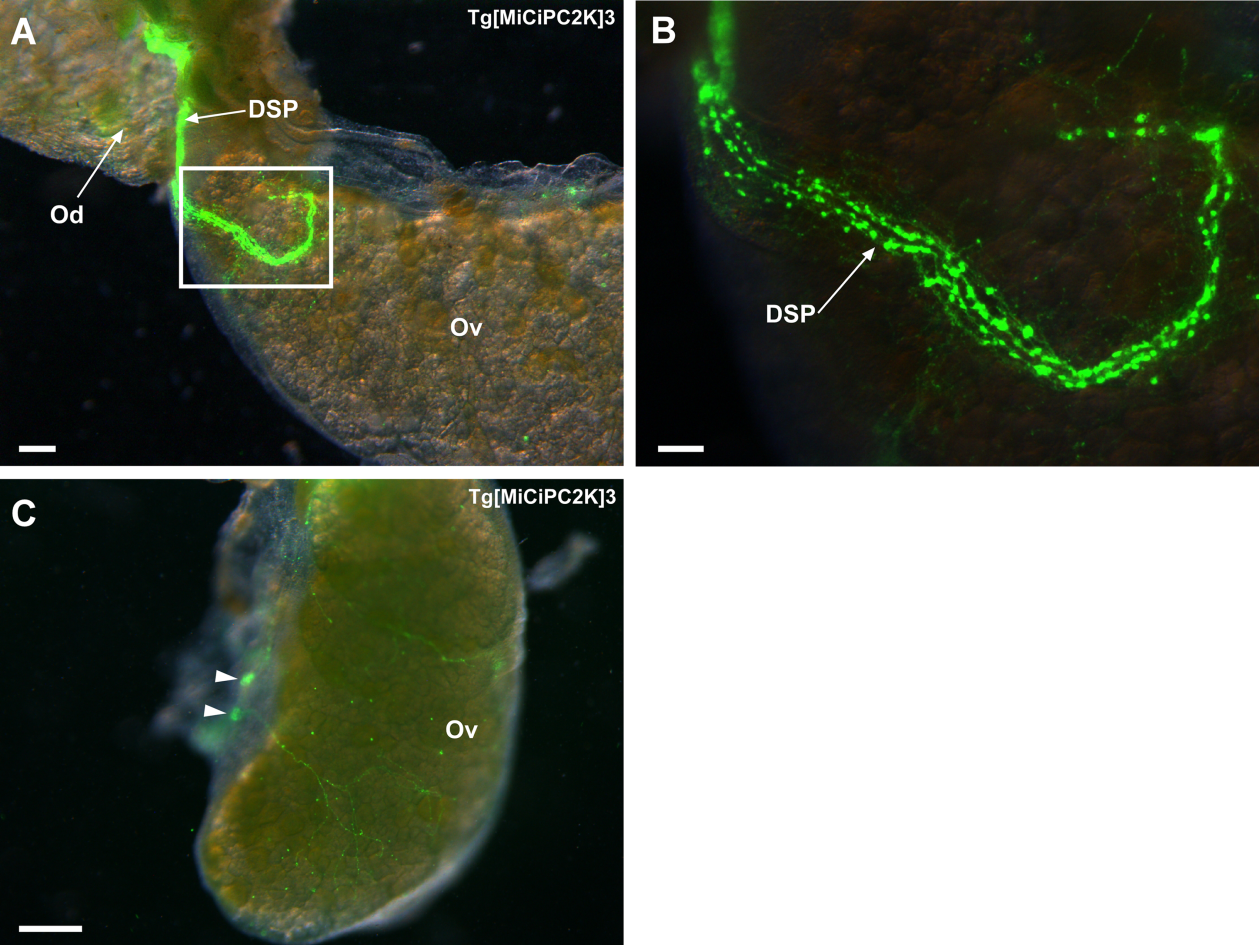
**(A) Innervation of the dorsal strand plexus to the ovary.** The image was obtained by the fluorescence stereo microscope. The dorsal strand plexus reaches the junction between the oviduct and the ovary. **(B) Magnified image of the rectangle region in (A).** The numerous neurons and innervation are observed at the junction between the oviduct and the ovary. **(C) View from the opposite side to the termination site of the dorsal strand plexus.** Nerves from the dorsal strand plexus innervate around the ovary. Neuron-like cells on the surface of the ovary are indicated by arrowheads. Od, oviduct; Ov, ovary; DSP, dorsal strand plexus. Scale bars indicate 600μm in (A), 200μm in (B), and 1 mm in (C).

We observed that the dorsal strand plexus reaches the junction between the oviduct and the ovary (Fig 7A). This observation is in part consistent with the previous immunohistochemistry using several anti-GnRH antibodies [15,50,52,56]. Furthermore, the innervation was observed not only at the junction but also around the whole ovary (Fig 7B and 7C). Some neuron-like cells were also detected on the surface of the ovary (Fig 7C). The detailed innervation of the dorsal strand plexus to the ovary indicates that the ovary is regulated by the materials secreted from the neurons of the dorsal strand plexus in *Ciona*.

In our previous study, more than thirty neuropeptides were identified from the central nervous system of adult *Ciona* [16,21]. Among these peptides, *Ciona* tachykinin-I (Ci-TK-I) enhanced oocyte growth by inducing expression of proteases including cathepsin D, chymotrypsin, and carboxy-peptidase B1 in the ovary [26,27]. Most of the identified neuropeptides including Ci-TK-I were expressed exclusively in the cerebral ganglion but not in the ovary, whereas many of their receptors were expressed in the ovary [16,21,26,27]. Combined with these findings, the present study suggests that the neuropeptides produced in the cerebral ganglion are secreted to the ovary *via* the dorsal strand plexus and/or visceral nerves, and regulate the oocyte growth and maturation.

In conclusion, we have identified a wide range of innervation patterns of the siphon and dorsal nervous system of adult *Ciona* between the cerebral ganglion and the periphery. Investigation of innervation to other tissues, such as stomach, heart, gill, and endostyle by the ventral nervous system is currently in progress. Identification of afferent and efferent nerves will reveal more rigorous neural network in adult *Ciona*. In addition, the immunohistochemistry and photoconverting of Kaede in the transgenic animal will allow us to further understand the peptidergic regulatory network of *Ciona*. These studies will also provide insights into the evolutionary process of neuroendocrine system in chordates.

## Supporting information

S1 Fig Variation of the innervation pattern of anterior nerves to the lobes of oral siphon. (A) The lobes numbered by 1 to 3 were innervated by multiple anterior medial nerves. (B) The ventral lobe numbered by 6 appeared to be innervated by ALN1a and ALN2a. Arrowheads indicate tentacle row. AMN, anterior medial nerve; ALN, anterior lateral nerve; CG, cerebral ganglion; OS, oral siphon. Scale bars indicate 2.5 mm.
(TIF)

S2 Fig Serial sections of the posterior region of the cerebral ganglion. Tg[MiCiPC2K]3 line was used to obtain images. Sixteen serial sections are tiled and numbered. Two posterior nerve are indicated by arrows. Two visceral nerves are indicated by arrowheads. The neurons of the dorsal strand plexus are also seen around the visceral nerves. CG, cerebral ganglion. Scale bars indicate 50μm in all images.
(TIF)

S3 Fig Front view of the posterior cerebral ganglion, dorsal strand plexus, and visceral nerves. Tg[MiCiPC2K]2 line was used to obtain images. The dark field image, bright field image, and superimposed image are shown in (A)-(C). The branching point of the VN2 is indicated by an arrowheads. The fibrous tissue lies above the branching point of the VN2. CG, cerebral ganglion; DSP, dorsal strand plexus; PN, posterior nerve; VN, visceral nerve. Scale bars indicate 500μm in all images.
(TIF)
