# Supplementary figures and images for: The nervous system of the adult ascidian Ciona intestinalis Type A (Ciona robusta): Insights from transgenic animal models
Source: PLoS One. 2017 Jun 26;12(6):e0180227. doi: 10.1371/journal.pone.0180227 (PMC5484526; doi:10.1371/journal.pone.0180227)

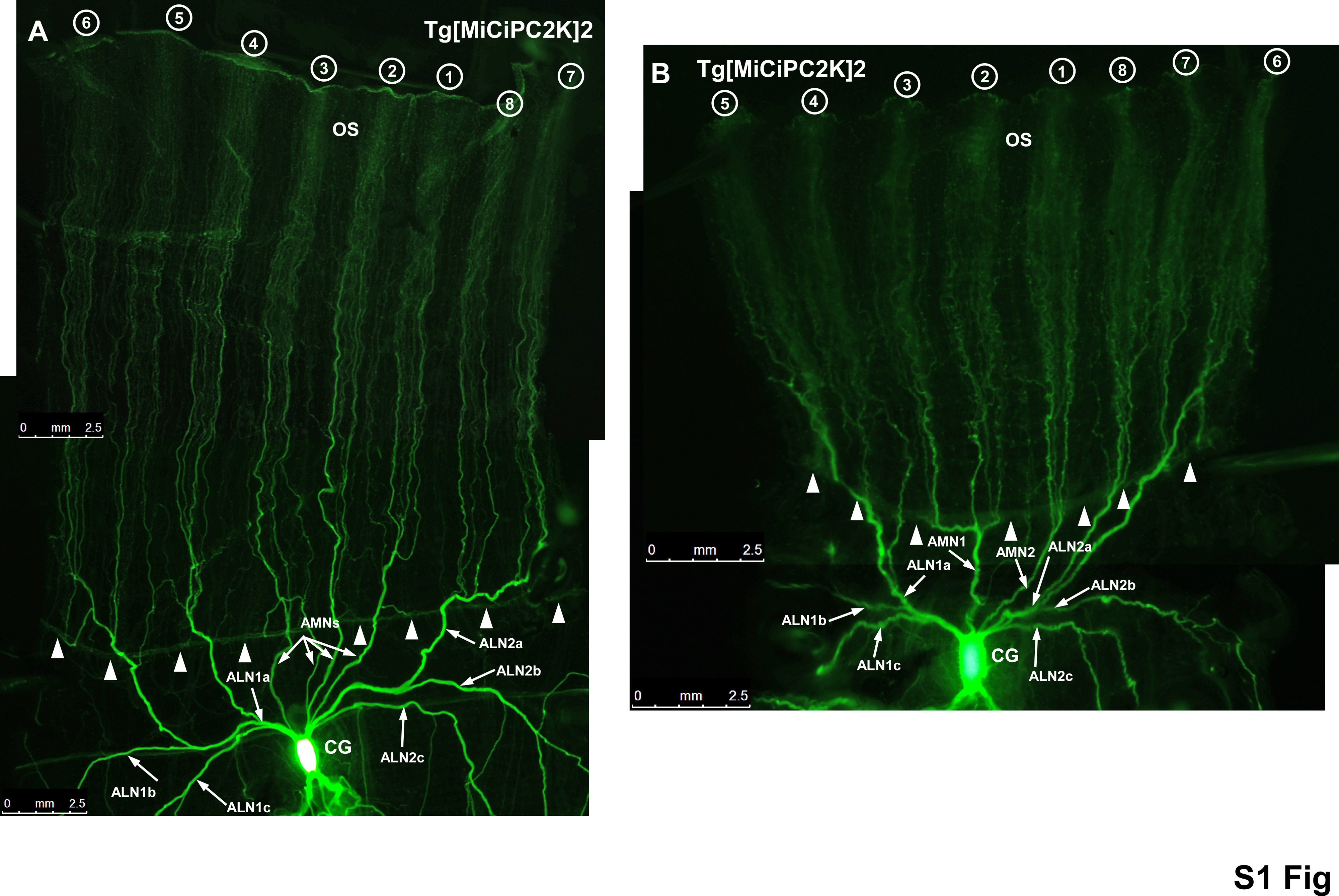

Supplement: S1 Fig — (A) The lobes numbered by 1 to 3 were innervated by multiple anterior medial nerves. (B) The ventral lobe numbered by 6 appeared to be innervated by ALN1a and ALN2a. Arrowheads indicate tentacle row. AMN, anterior medial nerve; ALN, anterior lateral nerve; CG, cerebral ganglion; OS, oral siphon. Scale bars indicate 2.5 mm. (TIF) [file pone.0180227.s001.tif]

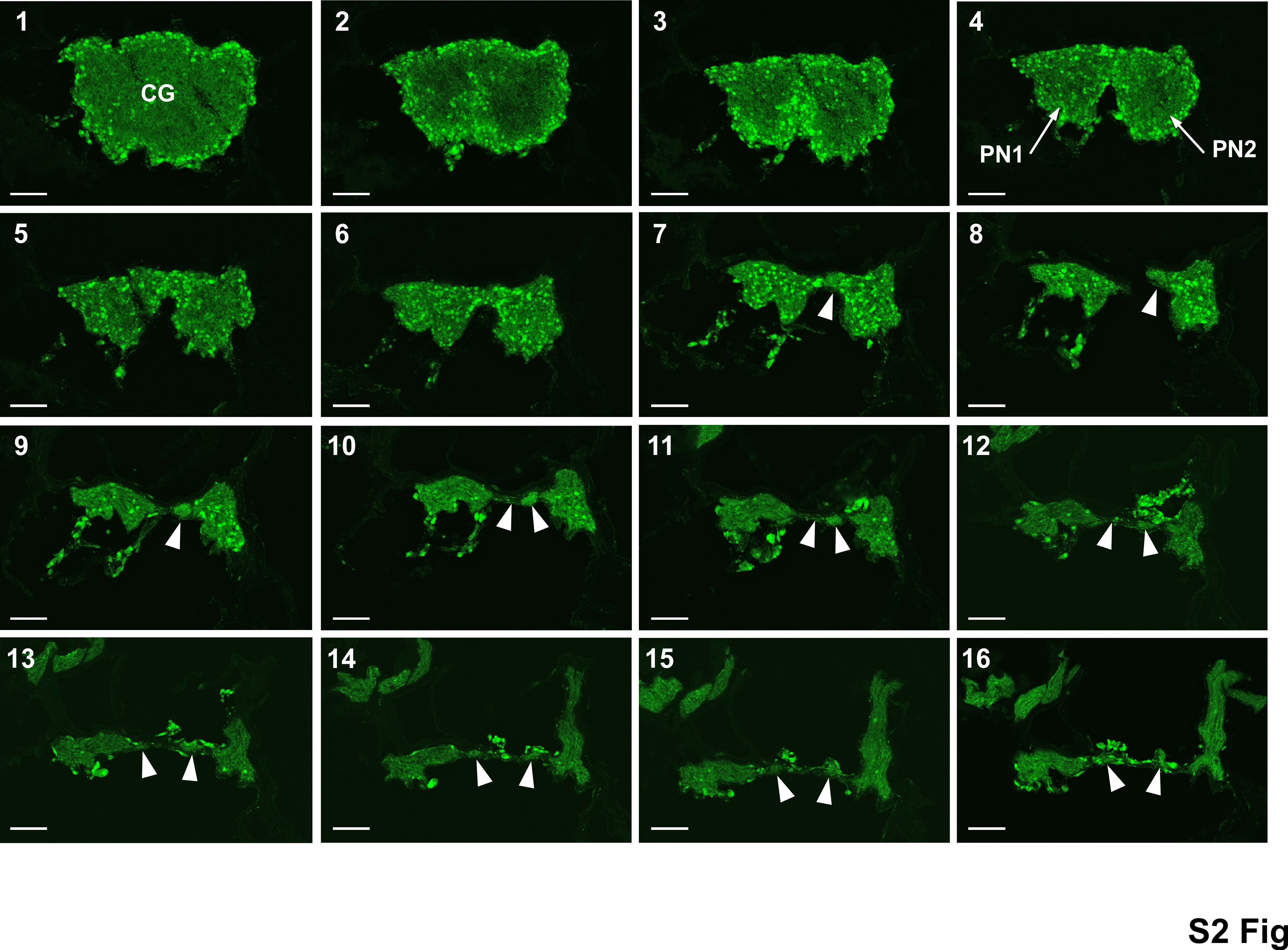

Supplement: S2 Fig — Tg[MiCiPC2K]3 line was used to obtain images. Sixteen serial sections are tiled and numbered. Two posterior nerve are indicated by arrows. Two visceral nerves are indicated by arrowheads. The neurons of the dorsal strand plexus are also seen around the visceral nerves. CG, cerebral ganglion. Scale bars indicate 50μm in all images. (TIF) [file pone.0180227.s002.tif]

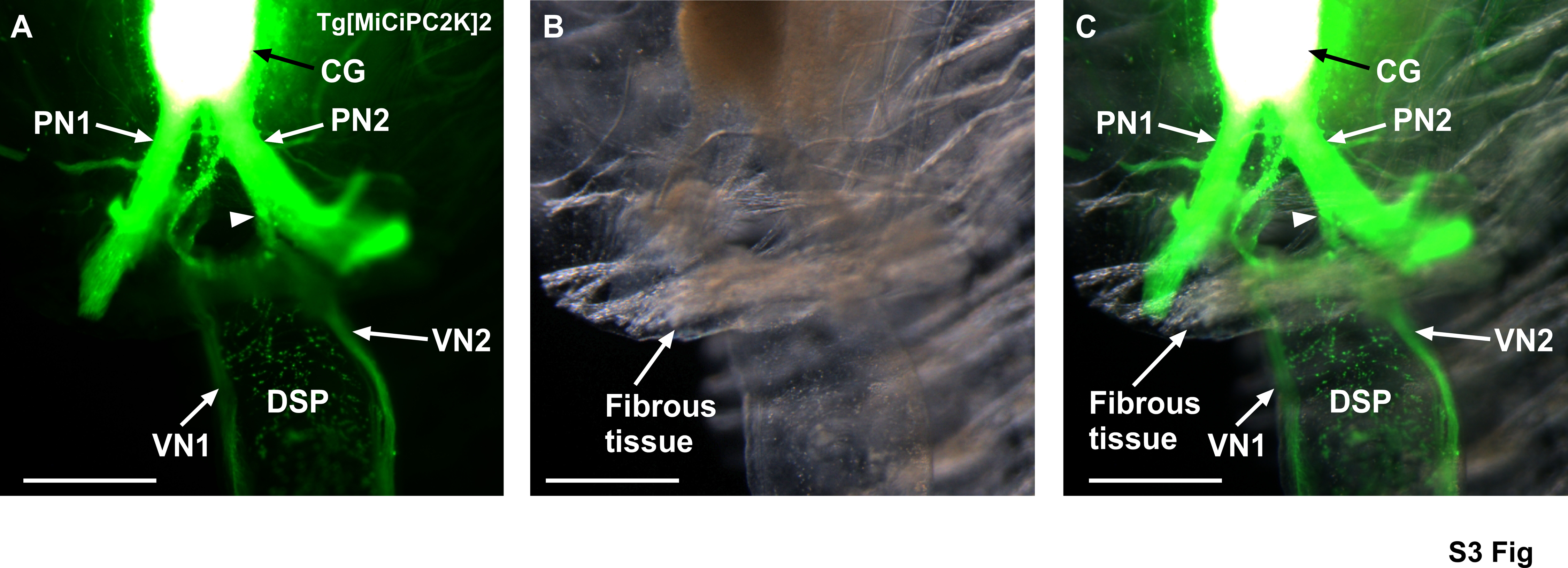

Supplement: S3 Fig — Tg[MiCiPC2K]2 line was used to obtain images. The dark field image, bright field image, and superimposed image are shown in (A)-(C). The branching point of the VN2 is indicated by an arrowheads. The fibrous tissue lies above the branching point of the VN2. CG, cerebral ganglion; DSP, dorsal strand plexus; PN, posterior nerve; VN, visceral nerve. Scale bars indicate 500μm in all images. (TIF) [file pone.0180227.s003.tif]
